# Hybrid Perovskite‐Photovoltaic and Solar‐Thermal Harvesting

**DOI:** 10.1002/advs.202509692

**Published:** 2025-09-24

**Authors:** Gan Huang, Parth H. Arya, David B. Ritzer, Nada A. Alati, Bahram Abdollahi Nejand, Ulrich W. Paetzold, Bryce S. Richards

**Affiliations:** ^1^ Institute of Microstructure Technology Karlsruhe Institute of Technology Hermann‐von‐Helmholtz‐Platz 1 76344 Eggenstein‐Leopoldshafen Germany; ^2^ Light Technology Institute Karlsruhe Institute of Technology Engesserstrasse 13 76131 Karlsruhe Germany

**Keywords:** heating, perovskite, photovoltaics, PV/T, solar

## Abstract

Single‐junction photovoltaics have inherent limitations as low‐energy photons below bandgap cannot generate electrical power, wasting solar energy. Here, a hybrid solar energy harvesting concept is presented. The prototype's core component consists of a semi‐transparent perovskite solar module that converts high‐energy solar photons into electrical power at 14.3% efficiency, while directing ≈55% of the low‐energy photons below bandgap to a solar‐thermal collector. The prototype can achieve an overall exergy efficiency of ≈30.0%, with exergy referring to the usefulness of transformed solar energy and its potential to do work. This high efficiency comes from 1) photovoltaic conversion, 2) low‐temperature heat generation (60 °C) from photovoltaic thermalization losses, and 3) high‐temperature heat generation (900 °C) from the low‐energy photons in a modeled large‐scale high‐concentration‐ratio solar concentrator. The exergy efficiency can advance to >40% if using a record‐efficiency perovskite photovoltaic. Overall, this study provides a high‐efficiency solution for harvesting the full solar spectrum.

## Graphical abstract

This study presents a hybrid solar energy harvesting concept that integrates a semi‐transparent perovskite solar module with a thermal collector. High‐energy photons generate electricity at 14.3% efficiency, while ≈55% of low‐energy photons provide heat. The system achieves ≈30% exergy efficiency, with modeling indicating >40% potential, offering a high‐efficiency route for full‐spectrum solar utilization.

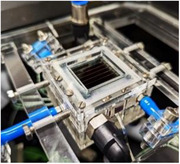

## 1 Introduction

Heat and electrical power are the primary energy end‐uses, accounting for ≈50% and 20% of global final energy consumption,^[^ ^1^ ^]^ respectively. However, the majority of heat (>85%) and electrical power (>60%) is still generated using fossil fuels, contributing to ≈50% of global carbon dioxide (CO_2_) emissions.^[^ ^1^, ^2^ ^]^ Industrial sectors alone consume around half of the global thermal energy.^[^ ^1^, ^2^ ^]^ These needs extend from low‐temperature heat (60–100 °C) and encompass medium‐temperature heat (100–400 °C), to even high‐temperature heat (>400 °C).^[^ ^1^ ^]^ Given these statistics, it is pivotal to establish a pathway for transforming both heat and electricity generation from fossil fuels to zero‐carbon sources in order to mitigate energy‐related CO_2_ emissions and limit climate change.

Solar photovoltaics (PV) technologies have undergone significant development in recent decades.^[^ ^3^ ^]^ However, solar cells intrinsically harvest only a limited share of the solar irradiation due to inherent thermalization and transparency losses, i.e., common silicon PV cells can only utilize photons of energy larger than the bandgap (photon energy higher than ≈1.1 eV, or wavelengths shorter than ≈1100 nm).^[^ ^4^, ^5^, ^6^ ^]^ Therefore, the PV power conversion efficiency (PCE) of the most common commercially‐available silicon‐based PV modules is typically in the range of 15–24%. The PCE of a single‐junction silicon solar cell is theoretically limited to 29.4% under standard test condition,^[^ ^4^ ^]^ while the remaining part of solar energy that cannot be converted into electrical power dissipates as waste heat in the device with thermalization losses accounting for 40–50% and transparency losses for 20–30%,^[^ ^5^ ^]^ not only underutilizing the incident solar irradiation, but also incurring a considerable reduction in PCE in the solar cell at increased operating temperatures.

Despite the possibility of recovering the waste heat from PV modules through the thermally‐coupled integration of underlying heat exchangers,^[^ ^7^, ^8^, ^9^ ^]^ these cells still encounter challenges associated with overheating when aiming for higher‐temperature thermal output from the thermally‐coupled heat exchangers.^[^ ^10^ ^]^ Consequently, the maximum temperature of the primary heat carrier in thermally‐coupled heat exchangers is limited to 60–85 °C as this is the common operation temperature of a solar cell.^[^ ^11^, ^12^ ^]^ However, such temperature limitation hinders the value of the heat. High‐temperature heat generation is particularly valuable for certain applications in industrial processes, where low‐grade heat cannot meet the necessary thermal requirements. Energy comprises both exergy and anergy, with exergy representing the quality of energy. While the electrical energy generated by PV modules has 100% exergy, the exergy of the heat (thermal energy) recovered by the heat exchanger is limited due to the restricted temperature range. Therefore, the integration of thermally‐coupled heat exchangers with PV modules, as a thermal management method, falls short in fully maximizing the potential of incident sunlight. Recently, spectral‐splitting techniques have been found to be effective solutions for spectrally separating the incident solar spectrum into distinct parts and separately directing them to PV modules and solar thermal (ST) absorbers for co‐generating electrical power and high‐temperature heat.^[^ ^13^, ^14^, ^15^ ^]^ However, a challenge of conventional spectral‐splitting techniques that persists is the occurrence of substantially increased system complexity and optical losses due to the necessary integration of additional optical filters.^[^ ^16^ ^]^ Addressing this challenge is crucial for effectively making the most of the entire solar spectrum. However, it also presents an opportunity to develop emerging hybrid solar harvesting methods.

Here, we present a solution for hybrid solar harvesting methods using semi‐transparent perovskite modules capable of effectively converting solar irradiation of energy above the bandgap to electrical power and selectively redirecting sub‐bandgap infrared photons of the solar spectrum toward an ST absorber. We fabricate wide‐band perovskite modules with an energy bandgap (*E*_g_) of ≈1.6 eV, which exhibit the ability to transmit or reflect a significant larger portion of the infrared solar spectrum (> 780 nm) toward the ST absorber,^[^ ^17^ ^]^ compared to other types of PV materials such as the common Si (>1100 nm) and GaAs (>900 nm).^[^ ^18^, ^19^ ^]^ This unique characteristic makes them highly suitable, as the perovskite modules efficiently generate electrical power while the ST absorber absorbs and utilizes the filtered‐out sub‐bandgap photons that would otherwise be lost.

## 2 Results

### 2.1 Hybrid Perovskite‐Photovoltaic and Solar‐Thermal (PVST) Concepts

**Figure 1:**
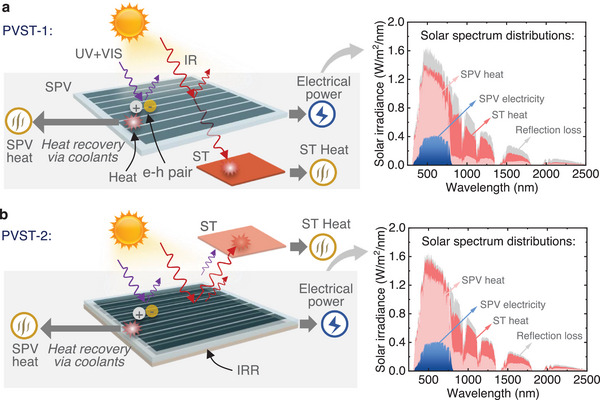
Schematic illustration of the concept of hybrid perovskite‐photovoltaic and solar‐thermal (PVST) harvesting. a) Concept of hybrid PVST based on selectively‐transmissive semi‐transparent perovskite photovoltaics (SPV). Most of the ultraviolet (UV) and visible (VIS) spectrum is absorbed by the SPV to excite electron‐hole (e‐h) pairs for electricity generation, and unavoidably generating heat in the SPV. The waste heat in the SPV can be recovered by attaching a heat exchanger below the SPV with flowing coolants (e.g., gas). A part of the infrared (IR) solar spectrum passes through the SPV and is then absorbed by a solar‐thermal (ST) absorber for high‐temperature heat generation. The solar spectrum can be effectively split at the wavelength ≈780 nm. We refer to this type of concept or design as PVST‐1. b) Concept of hybrid PVST design based on a selectively‐reflective perovskite component, i.e., a combination an SPV with an IR reflector (IRR). The waste heat in the SPV can be recovered by attaching a heat exchanger below the SPV with flowing coolants (e.g., gas or liquid). The IR solar spectrum is reflected by the IRR and then absorbed by an ST absorber. Both the reflected sunlight at the top surface of the SPV and the reflected IR light by the IRR can be absorbed by the ST absorber. We refer to this type of concept or design as PVST‐2. Of note is that the figures presented herein illustrate the underlying concepts rather than the detailed designs. Based on the outlined concepts, more details on the design and geometry will be provided and discussed in subsequent sections of this study.

Semi‐transparent PV modules based on emerging perovskite materials (organic‐inorganic lead halide perovskite APbX_3_) have aroused particular interest recently. The bandgap of perovskite solar modules is tunable from 1.5 to 2.3 eV by varying the chemical components. Perovskite solar modules thus can adjust the portion of sunlight being absorbed and transmitted and have therefore been utilized in various tandem solar module architectures. Perovskite solar modules have not only demonstrated a high PCE limit but also exhibit significant potential owing to their anticipated lower manufacturing costs in comparison to conventional silicon solar modules. To maximize the potential of the entire solar spectrum, we propose two‐types of hybrid perovskite‐photovoltaic and solar‐thermal (PVST) concepts, namely PVST‐1 in **Figure** **1**a, and PVST‐2 in Figure 1b.

In the PVST‐1 concept, photons with energies exceeding the bandgap energy in the ultraviolet (UV) and visible (VIS) spectrum are absorbed by the SPV, creating free charge carriers by exciting electrons from the valence band into the conduction band. It is important to note that there is still thermalization heat generated within the modules, as the in‐band sunlight cannot be fully converted to electricity. The heat generated per photon approximately corresponds to the excess energy per photon with respect to the bandgap. This waste heat can be captured by integrating an additional normal heat exchanger (not depicted in Figure 1) positioned beneath the SPV. This setup facilitates the conversion of SPV waste heat into additional useful low‐temperature heat, to increase the overall energy conversion efficiency. The SPV thus can generate both electrical power and heat, as shown in Figure . Incident photons with energies below the bandgap in the infrared (IR) spectrum will pass through the SPV and are subsequently absorbed by an ST absorber for heat generation. In an alternative concept for the PVST‐2 design, the SPV selectively absorbs high‐energy photons for electricity generation. An infrared reflector (IRR) is positioned at the bottom of the SPV, directing the IR photons toward a separate ST absorber. The difference in ST absorption between PVST‐1 and PVST‐2 is due to the non‐100% transmission and reflection below the perovskite bandgap, as observed in our experimental characterization in the next section.

The spectrum distributions in Figure 1 show that the incident solar spectrum is effectively separated into two distinct parts around the 780 nm wavelength. These components are then absorbed by the SPV and directed to the ST absorber, respectively. The PVST‐2 exhibits lower optical losses due to the recovery of the specular reflection optical loss within the system. The spectral distributions presented in Figure 1 provide evidence of the feasibility and efficiency of employing SPV in developing PVST solar collectors. The ST heat can reach high temperatures without affecting the SPV module, as it is thermally decoupled from the SPV module.

Perovskite solar cells, as a rapidly advancing photovoltaic technology, have been explored in hybrid photovoltaic‐thermal collectors for the co‐generation of electricity and heat. One common approach involves attaching a heat exchanger to the rear side of the perovskite module to recover thermal energy from waste heat,^[^ ^20^, ^21^ ^]^ similar to conventional photovoltaic‐thermal systems. However, in such configurations, the output temperature is limited to below ≈80 °C due to thermal coupling between the PV and thermal components. In another approach, to achieve higher thermal output temperatures, spectral‐splitting techniques have been applied using either thin‐film optical filters^[^ ^22^ ^]^ or liquid‐based optical filters^[^ ^23^ ^]^ to separate the solar spectrum between a perovskite module and a thermally decoupled absorber. These methods still rely on external optical filters, similar to traditional spectral‐splitting photovoltaic‐thermal designs.

In contrast, our study proposes a third, distinct method: we directly use semi‐transparent perovskite solar cells to spectrally split the incident solar radiation, to filter and redirect sub‐bandgap infrared photons to a separate solar‐thermal absorber, without the need for additional optical filters. Our earlier modelling work demonstrated the feasibility of this approach.^[^ ^17^ ^]^ In the present study, we take this concept further by combining experimental fabrication, optical and electrical characterization, exergy analysis, and system‐level modelling to realize and evaluate a functional hybrid PVST collector.

### 2.2 Prototype of Core Component: Semi‐Transparent Perovskite Photovoltaics (SPV), Infrared Reflector (IRR), and Solar‐Thermal Absorber (ST)

To validate the proposed PVST‐1 and PVST‐2 concepts, this section focuses on the preparation and characterization of the essential components: SPV, IRR, and ST. The SPV serves as the primary electro‐optical component, responsible for both generating electrical power and spectrally splitting the solar spectrum. Meanwhile, the IRR and ST components are designed to reflect and absorb the infrared spectrum, respectively.

**Figure 2:**
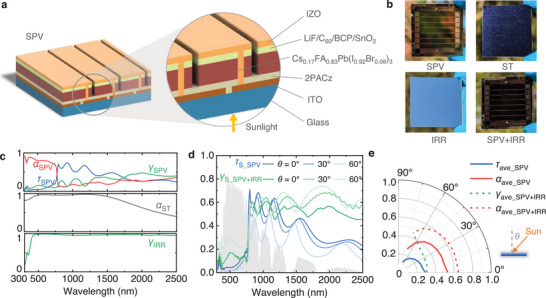
Optical properties of key components of SPV, IRR, and ST. a) Layer structure of SPV. b) Photographs of the front sides of SPV, ST, IRR, and SPV + IRR. c) Optical properties of SPV (absorptivity *α*_SPV_ and global transmittance *τ*_SPV_), ST (absorptivity *α*_ST_) and IRR (global reflectance *γ*_IRR_). SPV shows good selectively‐transmissive performance. d) Specular transmittance of SPV (*τ*_S_SPV_) and specular reflectance of SPV + IRR (*γ*_S_SPV + IRR_) for various incident angles. e) Spectrally‐weighted average transmittance and absorptance of SPV, and average reflectance and absorptance of SPV + IRR at the wavelength range of 0.3–2.5 µm.

The structure of the SPV fabricated in our lab involves layer deposition of glass/ ITO/ 2PACz/ Cs_0.17_FA_0.83_Pb(I_0.92_Br_0.08_)_3_/ LiF/ C_60_/ BCP/ SnO_2_/ IZO, as shown in **Figure** **2**a. Further information regarding the device fabrication process and materials preparation can be found in the Methods section and Figure S1 (Supporting Information). The SPV module retains its semi‐transparent appearance as shown in Figure 2b. Both the ST and IRR are commercially‐available materials, as detailed in the Method section. The effective area of the SPV, ST, IRR are standardized to 2 cm × 2 cm for comparative analysis and characterization purposes. The IRR is positioned directly beneath SPV to reflect the infrared spectrum without the use of adhesives.

The optical properties of SPV, IRR, and ST are fundamental and essential for analyzing the potential of PVST methods. The SPV absorptance (*α*_SPV_) exhibits a high spectrally‐weighted average value ≈ 0.79 for wavelengths shorter than ≈780 nm, as shown in Figure 2c. However, it sharply declines beyond this threshold, indicating that the bandgap of the SPV is approximately 1.6 eV. Conversely, the global transmittance of the SPV (*τ*_SPV_) shows a significant increase to 0.55 for wavelengths beyond 780 nm, allowing the infrared spectrum to pass through the SPV. Notably, the reflectance curve of the SPV reveals that ≈26.7% of the IR spectrum (>780 nm) is reflected by the SPV, due to the reflection loss at the TCO interfaces for the IR spectrum. The ST and IRR exhibit high absorptance and high reflectance for the solar spectrum, respectively. To minimize thermal cross‐talk between the SPV and ST units, heat transfer via conduction and convection can be suppressed by introducing vacuum gaps between the two components. Radiative heat transfer is further reduced by a spectrally selective coating on the surface of ST. This coating demonstrates a low mid‐infrared emissivity, as shown in Figure S2 (Supporting Information).

Under real‐world conditions, the incident angle of sunlight varies over time. Therefore, it is necessary to understand the optical properties of SPV at various incident angles. The specular transmittance of the SPV (*τ*_S_SPV_) decreases significantly as the incident angle increases from 0° to 60°, primarily due to increased reflection optical losses, as shown in Figure 2d. Comparatively, the SPV + IRR specular reflectance (*γ*_S_SPV + IRR_) is notably higher than the SPV specular transmittance (*τ*_S_SPV_) for wavelengths shorter than 780 nm and longer than 1200 nm, and thus can effectively direct more solar energy toward the ST absorber for heat generation. When *θ* = 0°, the average SPV + IRR out‐of‐band reflectance and SPV out‐of‐band transmittance are 0.56 and 0.54, respectively. At *θ* = 60°, these values change to 0.58 and 0.33, indicating that the SPV + IRR can direct more solar energy to the ST absorber at a large incident angle. The curves of *τ*_S_SPV_ and *γ*_S_SPV + IRR_ for more detailed angles can be found in Figure S3 (Supporting Information). The diffuse reflectance and transmittance of the SPV and the diffuse reflectance of the SPV + IRR are less than 5.0%, as shown in Figure S3 (Supporting Information). The spectrally‐weighted average values of SPV absorptance (*α*_ave_SPV_) and transmittance (*τ*_ave_SPV_), as well as SPV + IRR absorptance (*α*_ave_SPV + IRR_) and direct reflectance (*γ*_ave_SPV + ΙRR_) for the wavelength range of 0.3–2.5 µm, are calculated and presented in Figure 2e. The equation for calculating the spectrally‐weighted average values can be found in the Method section. The spectrally‐weighted average optical values of SPV and SPV + IRR for the wavelength range of 0.3–0.78 µm and 0.78–2.5 µm can be found in Figure S4 (Supporting Information). The optical properties of SPV and SPV + IRR demonstrate their effectiveness for the hybrid solar harvesting methods.

**Figure 3:**
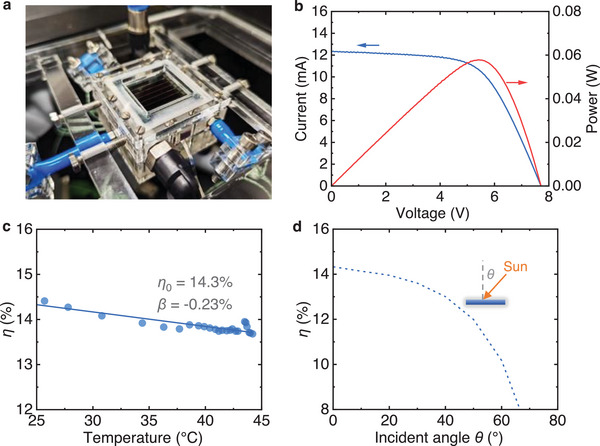
Photovoltaic performance of SPV. a) Photograph of the electrical testing platform. A nitrogen gas cooling channel is on the bottom of the SPV to control its operating temperature. b) Current‐voltage and power‐voltage curves of SPV at operating temperature 25 °C and under the normal incident angle. c) Photovoltaic power conversion efficiency of SPV as a function of operating temperature under the normal incident angle. d) Power conversion efficiency of SPV as a function of incident light angle.

In addition to the optical properties of the SPV, the electrical performance of the key component SPV is also critical to the design, which is evaluated using an indoor experimental setup, as depicted in **Figure** **3**a. To assess the impact of operating temperature on electrical efficiency, a cooling channel with flowing nitrogen is integrated beneath the SPV module. This setup allows for the modulation of the operating temperature.

Figure 3b presents the current‐voltage and power‐voltage curves of the SPV tested under a solar simulator at an operating temperature of 25 °C. The open‐circuit voltage and short‐circuit current are measured as 7.7 V and 12.3 mA, respectively. The power at the maximum power point is 57.6 mW, resulting in an electrical efficiency of 14.3% under the normal incident angle. Figure 3c illustrates the PCE of SPV under the normal incident angle as a function of its operating temperature. It is observed that the electrical efficiency decreases nearly linearly from 14.3% to 13.7% as the operating temperature rises from 25 °C to 44 °C, with a temperature coefficient of ‐0.23%/K. In Figure 3d, the electrical efficiency of the SPV is plotted as a function of the incident light angle. The SPV has a maximum efficiency of 14.3% at normal incidence but experiences a drop to below 10.2% at incident angles beyond 60°, as the reflection loss increases.

### 2.3 Solar Energy Conversion Process and Exergy Analysis of the Hybrid Perovskite‐Photovoltaic and Solar‐Thermal (PVST) Harvesting Methods

**Figure 4:**
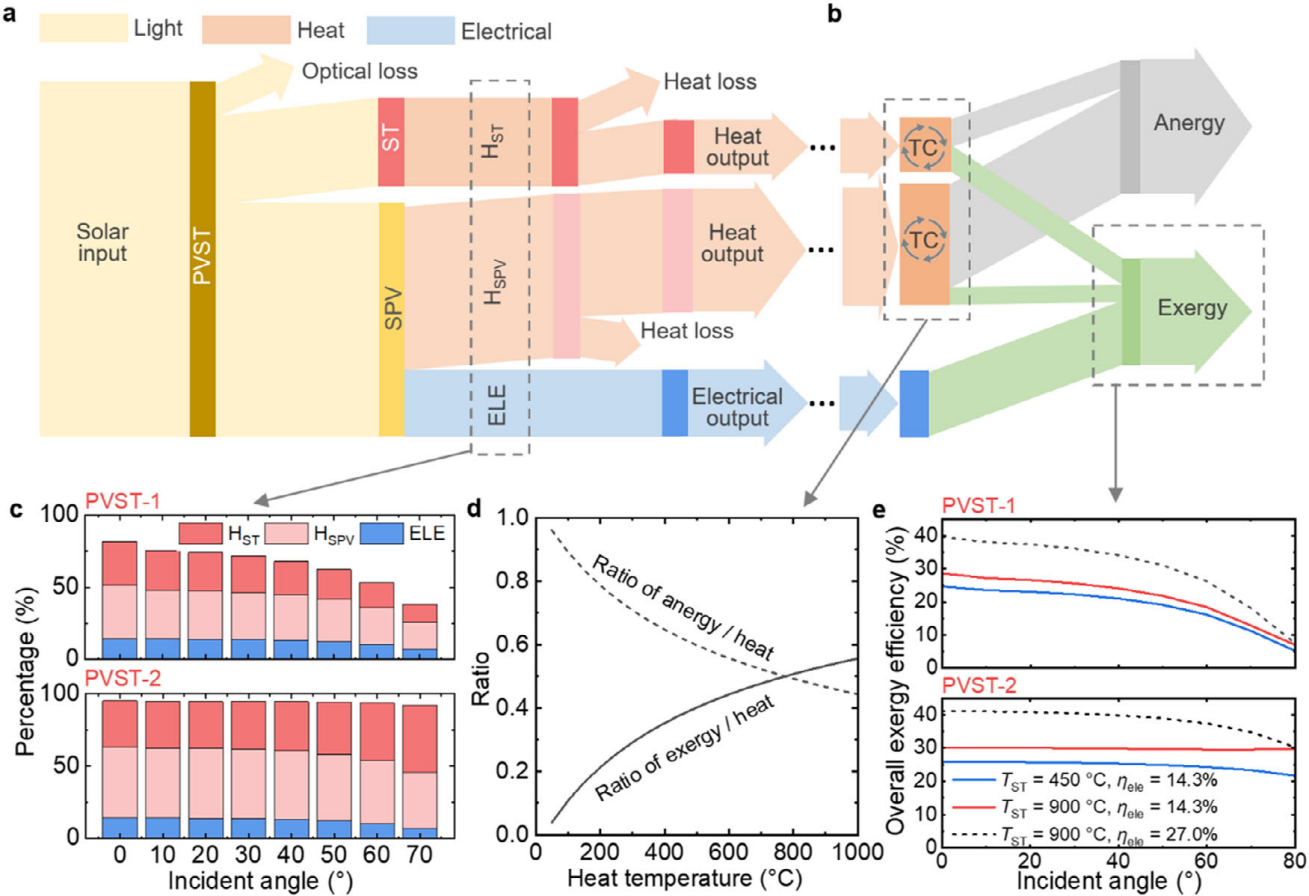
Solar energy conversion process and exergy analysis of hybrid perovskite‐photovoltaic and solar‐thermal (PVST) harvesting process. a) Solar energy conversion process of PVST. b) Exergy analysis for the outputs of heat and electrical power. The exergy output is the maximum amount of work that can be produced by the PVST process, which consists of electrical power output and the exergy portion in heat. c) The percentages of solar energy converted to electrical power (ELE), heat in SPV (H_SPV_) and heat in ST (H_ST_) in various sunlight incident angles for PVST‐1 and PVST‐2, when the SPV and heat temperatures are at ambient temperature, and without solar concentration. d) The ratio of exergy and anergy in heat as a function of the heat temperature. The ratio was calculated based on a thermodynamic‐cycle method as detailed in the Method section. The ST heat temperature can be increased via concentrating the sunlight, while the SPV heat temperature is fixed at 60 °C due to the operating temperature limit of the SPV module. e) The exergy efficiency of PVST‐1 and PVST‐2 processes for various hypothetical ST heat temperatures (450 and 900 °C) as a function of sunlight incident angle. Achieving these temperatures is feasible using high‐concentration‐ratio solar collectors.^[^ ^27^ ^]^ The overall exergy efficiency can be further improved when using the record‐efficiency perovskite cell (*η*_ele_ = 27.0%) in the literature.^[^ ^30^ ^]^

To analyze the performance and potential of PVST, we examine the solar energy conversion process and conduct a further exergy analysis. **Figure** **4**a illustrates the solar energy conversion process in PVST. The solar input can be converted into electrical power (ELE) and heat (H_SPV_) by SPV, and heat (H_ST_) by ST. The final heat outputs from SPV and ST should account for heat losses. It is important to note that the values of heat and electrical power differ. While electrical power is 100% exergy, the generated heat in parts is accounted as exergy (i.e., reversible energy) and anergy (i.e., waste heat). Only a part of heat can be converted into exergy based on thermodynamic cycles (TC) as shown in Figure 4b. The overall exergy is the maximum amount of work that can be produced by PVST, which consists of electrical power output and the exergy portion in SPV heat and ST heat.

The percentages of solar energy converted to ELE, H_SPV_, and H_ST_ for PVST‐1 and PVST‐2 are depicted in Figure 4c. PVST‐2 demonstrates overall better performance due to reduced system optical losses, particularly when the incident angle is large. The exergy ratio in heat is dependent on the heat temperature, which was calculated using a common thermodynamic‐cycle method as detailed in the Methods section. Figure 4d shows the ratio of exergy and anergy in heat as a function of the heat temperature. The overall exergy efficiencies of PVST‐1 and PVST‐2 are presented in Figure 4e. As stated above, the power generation of the perovskite PV module is measured experimentally. Additionally, the total heat generated by thermalization losses in the PV module is quantified. To analyze the exergy efficiency, we approximate the temperature of the low‐temperature heat carrier at 60 °C. This approximation is considered reasonable as it falls within the operating temperature range of the PV module and is a conservative estimate, given that temperatures as high as 80 °C have been demonstrated in the literature.^[^ ^7^, ^11^ ^]^ The perovskite material FAPbI_3_ has potential as a stable perovskite solar cells material between −40 and 75 °C.^[^ ^24^ ^]^ Partial substitution of FA⁺ with smaller‐radius inorganic cations such as Cs⁺ has been shown to further enhance thermal stability,^[^ ^24^, ^25^, ^26^ ^]^ e.g., Cs_0.17_FA_0.83_Pb(I_0.92_Br_0.08_)_3_ in this study. The operating temperature of the perovskite solar cells in our study (60 °C) falls well within this stable range. Further increasing the operating temperature decreases stability. Nevertheless, the impact of SPV temperature on the overall system performance is minimal, as shown in Figure S5 (Supporting Information). Similarly, the heat generation in the ST module is determined experimentally, and the temperature of the high‐temperature heat carrier needs to be approximated to evaluate the exergy efficiency. Since solar concentration techniques are applied to the ST module, operating temperatures in the range of 450 to 900 °C are achievable as demonstrated in the literature.^[^ ^27^, ^28^, ^29^ ^]^ Heat losses in concentrated ST collectors are typically below 10%.^[^ ^27^ ^]^ Although part of the solar spectrum is absorbed by the SPV module before reaching the ST module, the high temperatures of the ST module can still be attained by equivalently increasing the sunlight concentration ratio.

PVST‐2 exhibits a higher exergy efficiency potential compared to PVST‐1. The overall energy efficiency of PVST‐2 can reach 84.4% (broken down as 13.2% for SPV electrical power, 44.1% for SPV heat, 27.1% for ST heat), while the exergy efficiency of PVST‐2 can reach 30.0% (broken down as 13.2% from SPV electrical power, 2.4% from SPV heat, 14.4% from ST heat), when the high‐temperature heat carrier in ST reaches temperatures of 900 °C in a highly‐concentrated solar concentrator condition. In practical terms, this means that every 1 kWh of solar input can be converted into 0.13 kWh electricity, 0.44 kWh low‐temperature heat, and 0.27 kWh high‐temperature heat. This is equivalent to ≈0.3 kWh of useful work potential (comparable to electrical power). By employing the record‐efficiency perovskite cell (*η*_ele_ = 27.0% at 25 °C) reported in the literature,^[^ ^30^ ^]^ the overall exergy efficiency of PVST‐2 could potentially increase to >40% (broken down as 24.8% from SPV electrical power, 1.8% from SPV heat, 14.4% from ST heat. The purpose of using this record‐efficiency perovskite cell is to analyze the theoretical potential of our proposed PVST solar harvesting approach. The power conversion efficiency of the record‐efficiency perovskite cell is significantly improved from 14.3% (as reported for the perovskite cells used in this study) to 27.0%. This improvement primarily results from enhanced charge carrier collection and compositional optimization, which collectively contribute to a reduction in thermalization losses. The reduction in thermalization losses implies that less energy is converted into heat within the perovskite cell, thereby decreasing the thermal energy available for low‐temperature thermal harvesting. This reduction in generated low‐temperature heat has been appropriately considered in our exergy efficiency calculations. Regarding below‐bandgap transmission/reflection, we acknowledge that data for the record‐efficiency perovskite cell is not available in the NREL report or a publication. Therefore, we conservatively assume that the below‐bandgap transmission and reflection properties are the same as those of the perovskite cells used in this study, implying no improvement in this aspect. We recognize that achieving the theoretically predicted efficiency with our proposed PVST design will require continued research and development, including potential enhancements in below‐bandgap photon management and thermal energy recovery.

In certain industrial applications, the combination of electricity and heat is essential, such as in high‐temperature water electrolysis.^[^ ^31^ ^]^ Another potential method to enhance overall exergy efficiency is by raising the operating temperature of the ST module. However, the current temperature of 900 °C is already close to the typical temperature of ≈1000 °C for concentrated ST systems. Increasing the temperature further would significantly increase heat loss. For instance, at 1200 °C, heat loss rises to ≈20%,^[^ ^27^ ^]^ causing the overall exergy efficiency to remain at ≈40%, thereby negating any potential efficiency gains.

The exergy analysis results presented in Figure 4e are based on experimentally measured optical properties and the PCE of the perovskite solar module. In the current module design, there are still considerable spectral splitting losses, specifically the absorption and reflection of photons with wavelengths beyond 780 nm. As shown in Figure S6 (Supporting Information), the exergy efficiency of PVST‐1 can be improved from 28.6% to 36.6%, and that of PVST‐2 from 30.0% to 36.9%, if these spectral splitting losses were fully eliminated under ideal conditions. Even with a 50% reduction in these losses, the exergy efficiency could still be enhanced to 32.6% for PVST‐1 and 33.5% for PVST‐2.

The modelling methodology of solar energy conversion process and exergy analysis is detailed in the Method section. The analysis is based on the assumptions that the incident light is direct (i.e., the ratio of diffuse light in the incident light is zero), and that the optical loss in the solar concentrating process (e.g., optical losses caused by the shading of solar absorbers on solar reflectors) is ignored.

### 2.4 Modeled Performance of Large‐Scale Hybrid Perovskite‐Photovoltaic and Solar‐Thermal (PVST) Harvesting Collectors

**Figure 5:**
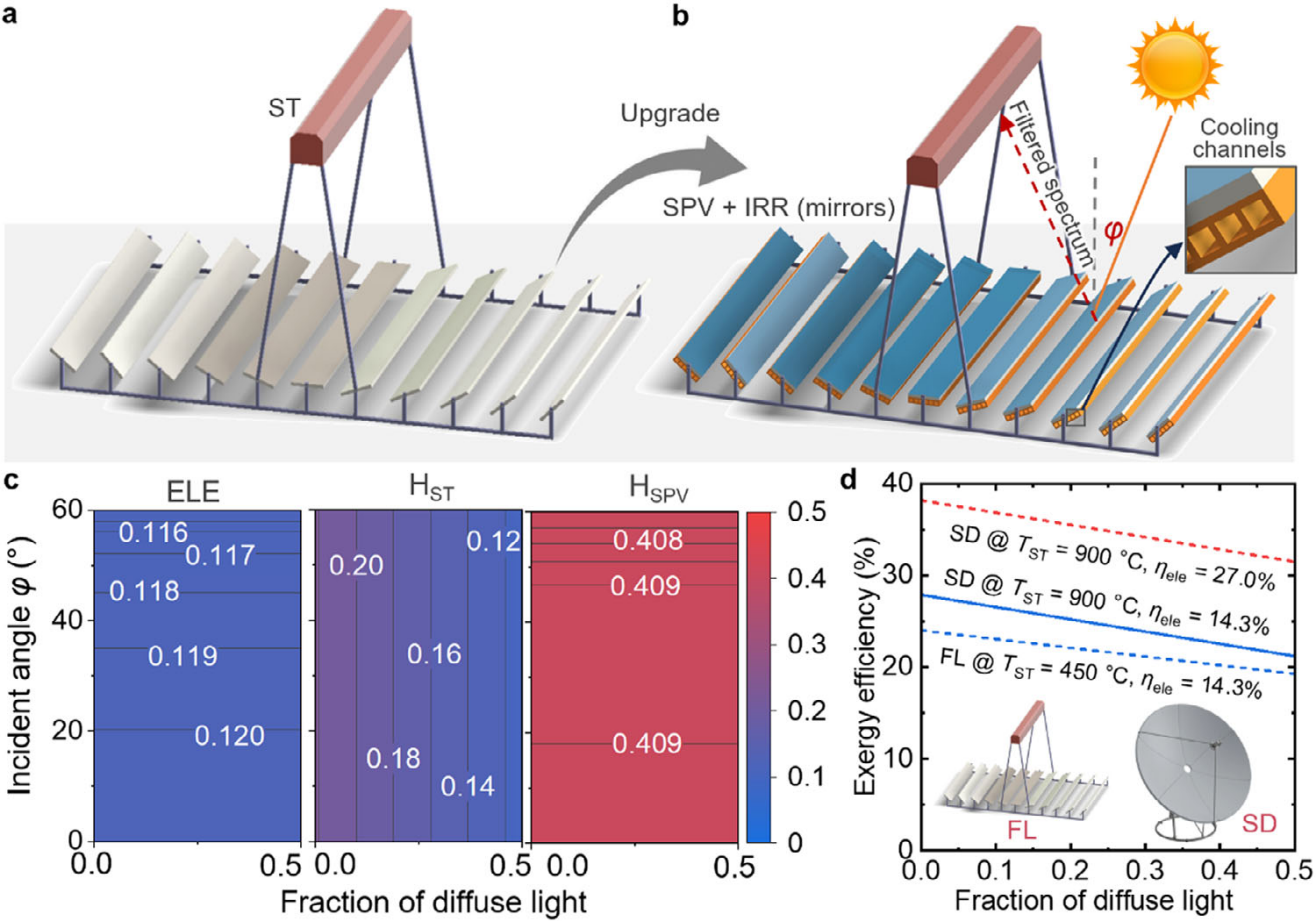
Design and performance of concentrated hybrid perovskite‐photovoltaic and solar‐thermal (PVST) collectors. a) A Fresnel‐lens (FL) solar concentrator. b) A hybrid PVST collector based on the PVST‐2 concept, which can be upgraded from the FL solar concentrator by attaching the SPV + IRR components above the mirrors of the FL. Regular heat exchangers with flowing coolants in the cooling channels can be attached below the SPV + IRR collector to recover waste heat from the PV modules. c) The percentages of solar energy converted to electrical power (ELE), heat in SPV (H_SPV_) and heat in ST (H_ST_) in various sunlight incident angles (*φ*) and fraction of diffuse light. d) Exergy efficiencies of hybrid PVST collectors based on FL and solar dish (SD) solar concentrators. FL solar concentrators can achieve 450 °C heat output, while SD solar concentrators can achieve a higher‐temperature heat output with 900 °C. The PCEs of the SPV in this study and the record‐efficiency SPV in the literature are 14.3% and 27.0%.^[^ ^30^ ^]^

To provide a more comprehensive analysis that considers real‐world factors like diffuse light and optical loss during the solar concentrating process, we propose a detailed mechanical design of a PVST solar collector and analyze its performance. In the commercially available Fresnel‐lens (FL) solar concentrator, depicted in **Figure** **5**a, sunlight is concentrated and absorbed by the ST collector to generate high‐temperature heat (details of the FL solar collector are provided in the Methods section). Understanding that the PVST‐2 concept can lead to a more efficient solar collector compared to the PVST‐1 concept in the above analysis, we design a hybrid solar collector based solely on the PVST‐2 concept. By integrating an SPV + IRR with the solar reflection mirrors, the conventional FL solar concentrator can be upgraded to a hybrid PVST solar collector, as shown in Figure 5b. The incident sunlight is spectrally separated by the SPV + IRR, which selectively absorbs the in‐band solar spectrum to generate electricity under non‐concentrated sunlight. The out‐of‐band solar spectrum is then reflected and concentrated onto the ST collector for high‐temperature heat generation. Additionally, regular heat exchangers can be attached below the SPV + IRR collector to recover waste heat from the PV modules, thereby generating additional low‐temperature heat. The SPV and ST components are physically separated and thermally decoupled by design, with the ST absorber positioned at a considerable distance from the SPV module. As a result, heat exchange between the components becomes negligible. We acknowledge that the additional costs associated with integrating heat exchangers should be carefully considered. The heat exchangers illustrated in Figure 5 can be based on commercially available designs widely used in conventional photovoltaic‐thermal collectors, which have demonstrated favourable payback times.^[^ ^32^ ^]^ Nevertheless, we recognize that the economic feasibility depends on various factors, including installation costs, market demand for heat and electricity, and regional energy prices.

The distribution of incident solar energy in the hybrid PVST solar collector, considering various incident sunlight angles (*φ*) and fractions of diffuse light, is shown in Figure 5c. The solar reflectors are assumed to track the sunlight in all cases to direct the sunlight onto the ST collector. The azimuth angle (*β*) is set to 0°. The modelling method is detailed in the Methods section. The fraction of diffuse solar irradiance relative to the global solar irradiance in the incident sunlight plays a crucial role in the performance of concentrating solar collectors. The advantage of the hybrid PVST solar collector in Figure 5b is that the SPV can effectively utilize both direct and diffuse light, as it operates under non‐concentrating conditions. Consequently, the PCE of the SPV remains unaffected by the fraction of diffuse light. However, the diffuse IR light reflected from the SPV + IRR cannot be utilized by the ST. Therefore, as the diffuse fraction increases, the proportion of solar energy used for generating heat by the ST decreases. The fraction of diffuse light varies depending on the location of the solar systems,^[^ ^33^ ^]^ as detailed in Figure S3 (Supporting Information). When the fraction of diffuse light is zero and the incident angle is zero, 12.0%, 20.4%, and 42.0% of the incident solar energy are converted to electrical power, heat in the SPV, and heat in the ST, respectively, resulting in an overall efficiency of 74.4%. Even when the fraction of diffuse light increases to 20%, the overall efficiency remains at 70.7%, indicating that the PVST collector can efficiently utilize diffuse light. The performance of the hybrid solar collector based on the PVST‐1 method is worse than that based on the PVST‐2 method, due to the more significant optical loss of PVST‐1 method.

The performance of PVST‐1 and PVST‐2 is also compared to a conventional existing concentrated solar thermal collector utilizing the Fresnel lens solar concentrator, as shown in Figure S7. The Fresnel lens solar concentrator achieves a peak thermal efficiency of 68%, which surpasses that of PVST‐1 but is lower than that of PVST‐2.

The exergy analysis in Figure 5d shows that the maximum exergy efficiency of the hybrid PVST solar collector is 24.0% when using the FL solar concentrator, with an achievable heat temperature of 450 °C and an SPV PCE of 14.3%. The overall exergy efficiency of the hybrid PVST solar collector increases to 27.9% at the hypothetical and achievable ST heat temperature of 900 °C (broken down as 12.3% from SPV electrical power, 2.2% from SPV heat, 13.4% from ST heat). Similar to the FL upgrading process, the conventional SD solar concentrator can also be upgraded to a hybrid PVST solar collector by integrating an SPV + IRR with the SD's solar reflection mirrors, as shown in Figure S8 (Supporting Information).

This study introduces a hybrid solar harvesting concept. There is still substantial room for enhancing the electrical performance of perovskite modules. Research groups worldwide are rapidly advancing perovskite solar technologies,^[^ ^34^, ^35^, ^36^, ^37^, ^38^, ^39^, ^40^ ^]^ opening exciting opportunities to incorporate higher‐efficiency SPV solar modules into our hybrid PVST design. By integrating these advancements, the overall performance and efficiency of the system can be significantly improved. Currently, efficiency‐record perovskite PV cells have achieved 27.0% efficiency.^[^ ^30^ ^]^ Utilizing the efficiency‐record perovskite cells, the overall exergy efficiency of the hybrid PVST solar collector could potentially reach 38.2% as shown in Figure 5d (broken down as 23.2% from SPV electrical power, 1.7% from SPV heat, 13.4% from ST heat). This study marks a significant step forward, with future work aimed at further enhancing and refining this approach to solar energy harnessing.

Specifically, we note that the PVST system incorporating the SPV module demonstrates higher exergy efficiency compared to existing single‐junction PV technologies, including the record‐efficiency silicon module and the highest‐performing perovskite module. Although multi‐junction solar cells—such as the three‐junction GaAs module—can achieve higher electrical efficiencies of up to 31.2%, the PVST system exhibits a theoretical exergy efficiency potential exceeding 40%, as discussed in the final section of the manuscript. This highlights the competitive advantage of the PVST concept in terms of total solar energy utilization.

## 3 Conclusion

This work presents a hybrid perovskite‐photovoltaic and solar‐thermal (PVST) solar energy harvesting method based on semi‐transparent perovskite photovoltaics (SPV). These hybrid PVST harvesting methods offer solutions for simultaneous electrical power generation and thermally decoupled heat generation, maximizing the potential of solar energy.

We demonstrate the feasibility and advantages of two types of hybrid PVST solar harvesting methods. The first type, the hybrid solar harvesting (PVST‐1) method, combines selectively transmissive SPV with a solar‐thermal (ST) collector, showing potential for electricity and heat co‐generation. The second type, the hybrid solar harvesting (PVST‐2) method, combines SPV and an infrared reflector (IRR), exhibiting better performance. The hybrid PVST‐2 method attains an overall exergy efficiency of ≈30.0% (broken down as 13.2% from SPV electrical power, 2.4% from SPV heat, 14.4% from ST heat). This efficiency is achieved when the heat generation of the high‐temperature heat carrier in ST is 900 °C in a modelled high‐concentration‐ratio solar concentrator.

Furthermore, this exergy efficiency has the potential to increase to >40% (broken down as 24.0% from SPV electrical power, 1.8% from SPV heat, 14.4% from ST heat) with the incorporation of current record‐efficiency perovskite cells. Further increasing the ST temperature cannot improve exergy efficiency, as it raises heat loss, eliminating any potential gains.

The proposed PVST architecture is conceptually extendable to large‐area systems. However, scaling introduces potential challenges related to both thermal and optical uniformity. On the thermal side, maintaining consistent cooling and effective heat extraction over larger SPV modules requires careful fluidic design, particularly to avoid temperature gradients that could impact both PV performance and material stability. These challenges can be mitigated by implementing optimized flow distribution strategies, such as using parallel serpentine channels, to ensure uniform coolant flow and effective heat removal across the entire surface. On the optical side, non‐uniform illumination caused by angular mismatch or imperfect concentrator alignment aberrations could lead to uneven power generation and localized heating. To minimize this, high‐precision solar tracking systems and optical homogenization techniques (e.g., secondary optics) may be employed. However, it should be noted that secondary optics may also introduce additional optical losses, presenting a trade‐off that warrants further investigation. Future studies should explore these aspects through large‐scale system integration and performance evaluation to assess thermal and optical uniformity under realistic operating conditions.

The outcomes of this study provide insights into the material performance characteristics and potential applications of hybrid PVST solar collectors. By advancing the understanding of their operating principles and improving material performance, such as power conversion efficiency (PCE) and thermal management, these systems hold great promise for contributing to a sustainable and clean energy future. The integration of hybrid PVST collectors into various sectors can pave the way for efficient solar energy utilization, reducing reliance on fossil fuels and mitigating climate change.

## 4 Experimental Section

### SPV, IRR, and ST Fabrication—Substrates, Solvents, and Materials for Perovskite Modules

The indium tin oxide (ITO) serves as the transparent front electrode due to its high conductivity and excellent visible light transmittance, essential for optimal SPV performance. The hole transport layer, 2PACz, follows, facilitating efficient hole extraction. The perovskite semiconductor, Cs_0.17_FA_0.83_Pb(I_0.92_Br_0.08_)_3_, absorbs photons above its bandgap and generates free charge carriers. The composition includes bromine to reduce ion migration and hysteresis,^[^ ^41^, ^42^ ^]^ while the double cation (Cs, FA) configuration enhances chemical and thermal stability for efficient operation under high irradiance conditions.^[^ ^43^, ^44^ ^]^ Lithium fluoride (LiF) acts as a passivation layer to minimize interface defects between the perovskite and the electron transport layer, C_60_. A buffer layer of BCP is then applied, followed by indium zinc oxide (IZO) as the transparent back contact, chosen for its high infrared transmittance (>1 µm), which is crucial for the ST component.^[^ ^45^ ^]^ Thereby, the functional layer stack of the SPV glass/ ITO/ 2PACz/ Cs_0.17_FA_0.83_Pb(I_0.92_Br_0.08_)_3_/ LiF/ C_60_/ BCP/ IZO is engineered to balance optical transparency and electrical efficiency. The starting materials are as follows: Soda‐lime glass substrates (polished, 1.1 mm thickness) were acquired with indium tin oxide (ITO) thin film (Luminescence Technology, CAS: 50926‐11‐9) and cut into a 30 × 30 mm^2^ format. Solvents were purchased from Sigma‐Aldrich: Chlorobenzene anhydrous 99.8% (CB, CAS: 108‐90‐7), Dimethyl Sulfoxide anhydrous ≥99.9%, Ethanol absolute 99.8%, Ethyl Acetate anhydrous 99.8%, N,N‐dimethylformamide ≥99.9% (DMF, anhydrous, 99.8%, CAS: 68‐12‐2). Materials and precursors were ordered from various suppliers: Cesium Iodide (CsI, abcr, CAS: 7789‐17‐5), Formamidinium iodide (FAI, Dyenamo, CAS: 879643‐71‐7), Fullerene‐C60 (C_60_, Sigma‐Aldrich, CAS: 99685‐96‐8), Lead Bromide (PbBr_2_, TCI, CAS: 10031‐22‐8), Lead Chloride (PbCl_2_, TCI, CAS: 7758‐95‐4), Lead iodide (PbI_2_, TCI, CAS: 10101‐63‐0), Lithium Fluoride (LiF, Sigma‐Aldrich, CAS: 7789‐24‐4), Fullerene‐C_60_ (C_60_, Sigma‐Aldrich, CAS: 99685‐96‐8), Methylammonium Chloride (MACl, Dyenamo, CAS: 593‐51‐1), 2PACz (TCI, CAS: 20999‐38‐6), Tetrakis(dimethylamino)tin (TDMASn) (99.99%‐Sn, PURATREM, CAS: 1066‐77‐9).

### SPV Module Fabrication

The perovskite solar submodules with the following configuration glass /  ITO  /  2PACz  /  Cs_0.17_FA_0.83_Pb(I_0.92_Br_0.08_)_3_  /  LiF  / C_60_  / BCP  / SnO_2_/ IZO were fabricated following the device fabrication process described below. The monolithic fabrication of interconnections (P1, P2, P3) between individual solar cells to create modules was performed via laser scribing utilizing a custom‐built laser scribing setup (Bergfeld Lasertech GmbH). The setup consists of a 1 ns Nd:YVO_4_ laser (Picolo AOT 10‐MOPA, InnoLas Laser GmbH) with 1064 and 532 nm, a camera for alignment, a scanner and an air filtering circuit. The sample compartment is integrated in a nitrogen‐filled glovebox to avoid the degradation of water‐ or oxygen‐sensitive layers. All laser scribing is performed at a laser wavelength of 532 nm wavelength and from the film side. First, the P1 scribing process to structure the transparent front contact (ITO) was carried out at a laser pulse fluence of 2 J cm^−2^ and a scribing speed of 50 mm s^−1^. The substrates were then cleaned, utilizing an ultrasonic bath with deionized water, acetone and isopropanol (10 min each), followed by a treatment with oxygen plasma (3 min). The solution for the hole transport layer was prepared by dissolving the 2PACz powder in Ethanol (1 mmol L^−1^). The layer was fabricated via spin‐coating by dropping the solution on the substrate at 3000 r.p.m. for 30s and annealing it for 10 min at 100 °C. The double‐cation perovskite precursor solution Cs_0.17_FA_0.83_Pb(I_0.92_Br_0.08_)^3^ was prepared by dissolving 0.12 mmol PbBr_2_ (46 mg), 0.17 mmol CsI (44 mg), 0.88 mmol PbI_2_ (444 mg, 10% excess of PbI_2_), 0.83 mmol FAI (143 mg) in a 1 mL solvent mixture of DMF:DMSO (4:1 volume ratio). Furthermore, 35 µL of a PbCl_2_:MACl solution dissolved in 1 mL DMSO with a molar ratio of 1:1 was added to the perovskite precursor solution as a bulk passivation additive. The solution was then deposited via two‐step spin coating process: 1) 1000 r.p.m. for 10 s (2000 r.p.m. s^−1^), 2) 5000 r.p.m. for 40 s (2000 r.p.m. s^−1^). 20 s before the end of the second spin coating step, 150 µL CB was dropped on the spinning substrate. The deposition of the perovskite absorber was finished by annealing the layer at 100 °C for 30 min in inert atmosphere. After annealing, 1 nm LiF as passivation layer and 23 nm of C60 as electron transport layer were deposited via thermal evaporation at evaporation rates of 0.1‐2 Å s^−1^ and a pressure of 10^−6^ mbar. This was followed by an atomic layer deposition of 35 nm SnO_2_ via 300 cycles. Subsequently, the P2 lines were laser scribed with a laser pulse fluence of 0.4 J cm^−2^ and a scribing speed of 33 mm s^−1^ to enable a contact between front and back electrode. As a transparent back electrode, indium‐doped zinc oxide (IZO) with a thickness of 165 nm was deposited via sputtering (Kurt J. Lesker PVD‐75). To finish the devices, the P3 lines were laser scribed at a laser pulse fluence of 0.3 J cm^−2^ and a scribing speed of 100 mm s^−1^.

### IRR and ST Fabrication

Both the ST and IRR materials used in this study are commercially available products purchased from Alanod Solar GmbH in Germany. The ST material consists of a solar‐absorbing coating applied to an aluminum substrate, specifically designed to convert solar energy into heat efficiently. The IRR material is composed of polished aluminum, serving as a reflective layer to enhance the system's overall efficiency. The detailed fabrication processes of the ST and IRR materials are proprietary and not publicly disclosed by the company. However, these materials are widely used and considered cost‐effective in the field of solar‐thermal technologies. For our experimental setup, the ST and IRR materials were cut to the desired size using a foil cutter. The SPV + IRR configuration was assembled by attaching the IRR to the bottom of the SPV to improve the thermal and optical performance of the system.

### Optical Characterization

The measurements of the reflection and transmission properties were carried out using UV–vis–NIR Spectrophotometer (Agilent Cary 7000) across wide range of wavelengths (300–2500 nm) with a dual light source consisting of a quartz tungsten halogen visible/NIR and deuterium arc for the UV. The instrument was equipped with an integrated sphere with a highly reflective inner surface (Spectralon) to collect both diffused and direct light. Direct reflectance and transmittance at variable angles were measured by the Cary 7000 UMS module. The measurements were recorded and controlled via Cary WinUV software. The spectrally‐weighted values in the wavelength range of a<*λ*<b is calculated by:

(1) $$ATR_{\mathrm{ave}}=\int_{a}^{b}ATR\left(\lambda \right)G_{\mathrm{AM}1.5}\left(\lambda \right)d\lambda /\int_{a}^{b}G_{\mathrm{AM}1.5}\left(\lambda \right)d\lambda$$

where *G*_AM1.5_ is AM1.5 standard solar spectrum. *ATR*(λ) is the spectral optical property (absorption, transmittance or reflectance as a function of wavelength) of a sample.

### Electrical Characterization

The electrical performance of the perovskite solar module was evaluated using a continuous dual‐lamp solar simulator (class AAA) from Oerlikon and high‐precision source meter (Keithley 2000). To simulate sunlight intensity and spectral distribution, a combined dual xenon lamp (Wacom model KXL‐500F) and halogen lamp (Ushio model JC‐36V‐400 W) were used as the light source. An IR temperature sensor was used to measure the operating temperature of the solar module.

### Calculation of the Spectral Distribution for SPV Electricity and Heat

The wavelength‐dependent electrical power generation of the SPV module was determined using the measured external quantum efficiency (EQE) spectrum of a SPV reference solar cell with the same perovskite composition and bandgap, and a similar performance as the SPV module. This EQE spectrum was multiplied by the photon flux at the corresponding wavelengths of the AM1.5G irradiance spectrum as well as by the electron charge to obtain the resulting short‐circuit current density (*J*_sc_) per cm^2^ and per wavelength. To determine the generated electrical power per cm^2^ and per wavelength of the SPV module, we multiplied this current density by the measured characteristic values of the SPV module, specifically the open‐circuit voltage (*V*_oc_) and fill factor (*FF*). Finally, the generated heat of the perovskite solar cell was calculated by subtracting the generated electrical power per cm^2^ and per wavelength and from the total absorbed irradiance of the perovskite layer. It is important to note that in this representation of perovskite solar cell heat generation, we have neglected the small fraction of radiative recombination losses, as they are minimal compared to other loss channels that result in heat.

### Solar Conversion Process and Exergy Analysis Methodology

Exergy is a fundamental concept in the field of thermodynamics and engineering. The value of heat depends on its temperature. A common way to assess the quality of heat at various temperatures is through exergy analysis. The heat consists of exergy (i.e., useful work potential) and anergy (i.e., waste heat). The ratio of exergy in heat, *ρ*_ex‐h_, is defined as:

(2) $$\rho_{\mathrm{ex}-h}=\frac{Q_{\mathrm{exe}}}{Q_{\mathrm{exe}}+Q_{\mathrm{ane}}}\;$$

where *Q*_exe_ is the exergy, and *Q*_ane_ is the anergy in heat. The sum of *Q*_exe_ and *Q*_ane_ is the total heat. The value of *ρ*_ex‐h_ depends on the heat temperature and also the environmental temperature, which can be calculated by:^[^ ^46^, ^47^, ^48^ ^]^

(3) $$\rho_{\mathrm{ex}-h}=\frac{\Delta h-T_{0}\Delta S}{\Delta h}$$

where Δ*h* and Δ*S* are the changes of enthalpy and entropy, and *T*_0_ is the environmental temperature. Both Δ*h* and Δ*S* depend on temperature and can be calculated by:^[^ ^46^, ^47^, ^48^ ^]^

(4) $$\Delta h=c_{p}\;\left(T_{\mathrm{out}}-T_{\mathrm{in}}\right)$$

(5) $$\Delta s=c_{p}\;\ln\frac{T_{\mathrm{out}}}{T_{\mathrm{in}}}$$

The value of *ρ*_ex‐h_ can thus be calculated by:

(6) $$\rho_{\mathrm{ex}-h}=1-\frac{T_{0}}{T_{\mathrm{out}}-T_{\mathrm{in}}}\ln\frac{T_{\mathrm{out}}}{T_{\mathrm{in}}}$$

where the *T*_out_ is the output heat temperature and *T*_in_ is the inlet (or initial) temperature of the heat carrier (e.g., heat transfer oils in ST absorbers). In this study, the *T*_in_ is the same as the environmental temperature (*T*_0_ = 300 K). The electrical power is 100% exergy. In the PVST process in Figure 4, the percentage of optical loss is based on the real characterization results of SPV and SPV + IRR. The percentage of heat loss of solar dishes is set to be 10% of the heat. The percentage of heat loss can be less than 10% when heat temperature is 450–900 °C.^[^ ^49^ ^]^ Therefore, the percentage of heat loss in the exergy analysis is assumed to be 10% as a conservative estimate. The analysis is based on the assumptions that the incident light is direct (i.e., the ratio of diffuse light in the incident light is zero), and that the optical loss in the solar concentrating process (e.g., optical losses caused by the shading of solar absorbers on solar reflectors) is ignored.

### Modelling Methodology of Hybrid PVST Solar Collectors

The geometric and optical properties of the FL mirrors and the ST collector in the PVST collector are the same as those in the commercial FL solar collector from *Industrial Solar GmbH* (model number: LH‐11),^[^ ^49^ ^]^ comprises a 7.5 m long and 4.1 m wide array of Fresnel mirrors, with the ST collector located 4.5 m above the ground. The aperture surface of mirrors is 23 m^2^. The optical efficiency of the Fresnel lens CST solar collector depends on the zenith angle (*φ*) and azimuth angle (*β*). According to the technical datasheet of *Industrial Solar GmbH*, the Fresnel lens CST solar collector has an overall optical efficiency of 0.68 (i.e., the thermal efficiency of the CST collector at ambient temperature) under sun in zenith (*φ* = 0°, *β* = 0°), which considers all the optical losses of the mirrors, ST absorber, and shading. The optical efficiency of the ST collector is set as 0.80, while the optical efficiency of the Fresnel concentrator is 0.85, thus yielding an overall optical efficiency of 0.8 × 0.85 = 0.68. The optical efficiency of the FL concentrator considers the optical loss due to the absorptance of the mirror, the gap between adjacent mirrors and the shadowing on the mirrors. The optical efficiency of the ST collector considers the reflection optical loss of the ST collector. The overall optical efficiency of the Fresnel lens CST solar collector is 0.68 when both the zenith angle (*φ*) and azimuth angle (*β*) are zero. The optical efficiency of the FL solar collector as functions of the zenith and azimuth angles provided by the manufacturer.^[^ ^49^ ^]^ In this study, we fix the azimuth angle to be zero, and vary the zenith angle in this study. Each Fresnel lens can independently rotate and track the sunlight, to ensure the reflected light can be directed to the focus point. The SPV can utilize both the direct and diffuse light, but only the direct light can be concentrated. The sharp decline in SPV performance at incident angles greater than 60° can be effectively mitigated through solar tracking. The incident angle of sunlight on the SPV module (θ) is determined by both the solar zenith angle (φ) and the reflection angle of the optical path (β), as illustrated in Figure S9 (Supporting Information). In our PVST‐2 solar tracking configuration, the maximum incident angle on the SPV remains below 58.9°, even when the solar elevation is as low as 10° (i.e., φ = 80°). This indicates that, under typical operational conditions, the incident angle will stay below 60°, thereby avoiding the sharp decline in SPV performance observed at higher angles. Based on the incident angles on components, we can then calculate the portions of the solar energy being absorbed, reflected and transmitted according to Figure 2f–h, for generating electrical power and heat. All the losses in the ST and Fresnel concentrator are considered in the simulation. The real measured properties of SPV + IRR units under different incident angles are also considered in the simulation. The SPV efficiency is assumed to be independent of the solar concentration ratio. In the solar dish solar concentrator, the optical efficiency of the ST collector is set as 0.9, while the optical efficiency of the dish reflector is 0.9, thus yielding an overall optical efficiency ≈ 80%.^[^ ^27^ ^]^ In our modelling, the temperature of the low‐temperature heat carrier is set at 60 °C. This approximation is reasonable as it falls within the operational temperature range of the PV module and represents a conservative estimate, especially considering that temperatures as high as 80 °C have been reported in the literature.^[^ ^11^, ^12^ ^]^ On the other hand, due to the application of solar concentration techniques to the ST module, much higher operating temperatures ranging from 450 to 900 °C are achievable, as demonstrated in previous studies.^[^ ^27^, ^28^, ^29^ ^]^ The percentage of heat loss is less than 10% in the Fresnel solar concentrator, when the heat temperature is 450 °C.^[^ ^49^ ^]^ The percentage of heat loss is less than 10% in the high‐concentration‐ratio solar dish solar concentrator, when the heat temperature is 900 °C.^[^ ^49^ ^]^ Therefore, the percentage of heat loss in the modelling is assumed to be 10% as a conservative estimate.

## Conflict of Interest

The authors declare no conflict of interest.

## Author Contributions

G.H. developed the concept and research methodology. G.H., P.H.A., and N.A.A. conducted the material characterization. D.B.R. and B.A.N. fabricated the perovskite solar cells. G.H. conducted data processing and solar collector performance analysis. All authors contributed to writing and revising the manuscript. G.H., B.S.R., and U.W.P. supervised the research and concluded the discussion of the results. G.H. directed this project.

## Supporting information



Supporting Information

## Data Availability

The data that support the findings of this study are available from the corresponding author upon reasonable request.
