# Supplementary material for: Hybrid Perovskite‐Photovoltaic and Solar‐Thermal Harvesting
Source: Adv Sci (Weinh). 2025 Sep 24;12(42):e09692. doi: 10.1002/advs.202509692 (PMC12622551; doi:10.1002/advs.202509692)
Supplement: Supplementary file 1 — Supporting Information [file ADVS-12-e09692-s001.pdf]

Supporting Information

Hybrid perovskite-photovoltaic and solar-thermal harvesting

Gan Huang[1, *], *Parth H. Arya*[1], *David B. Ritzer*[1, 2], *Nada A. Alati*[1], *Bahram Abdollahi Nejand*[1, 2], *Ulrich W. Paetzold*[1, 2], *Bryce S. Richards*[1, 2, *]

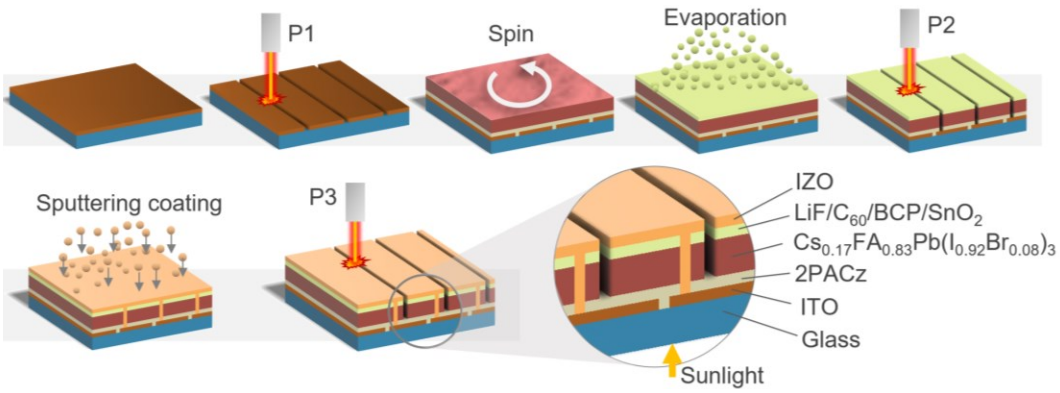

Figure S1. **The fabrication process of the semi-transparent perovskite module**. The module with the following configuration glass / ITO / 2PACz / $Cs_{0.17}FA_{0.83}Pb(I_{0.92}Br_{0.08})_3$ / LiF / C_{60} / BCP / SnO_2/ IZO were fabricated following the device fabrication process described below. The monolithic fabrication of interconnections (P1, P2, P3) between individual solar modules to create modules was performed via laser scribing utilising a custom-built laser scribing setup (Bergfeld Lasertech GmbH). The setup consists of a 1 ns Nd:YVO_4 laser (Picolo AOT 10-MOPA, InnoLas Laser GmbH) with 1064 nm and 532 nm, a camera for alignment, a scanner and an air filtering circuit. The sample compartment is integrated in a nitrogen-filled glovebox to avoid the degradation of water- or oxygen-sensitive layers. All laser scribing is performed at a laser wavelength of 532 nm wavelength and from the film side. First, the P1 scribing process to structure the transparent front contact (ITO) was carried out at a laser pulse fluence of 2 J/cm^2 and a scribing speed of 50 mm/s. The substrates were then cleaned, utilising an ultrasonic bath with deionised water, acetone and isopropanol (10 min each), followed by a treatment with oxygen plasma (3 min). The solution for the hole transport layer was prepared by dissolving the 2PACz powder in Ethanol (1 mmol/L). The layer was fabricated via spin-coating by dropping the solution on the substrate at 3000 r.p.m. for 30s and annealing it for 10 min at 100 °C. The double-cation perovskite precursor solution $Cs_{0.17}FA_{0.83}Pb(I_{0.92}Br_{0.08})$[3] was prepared by dissolving 0.12 mmol $PbBr_2$ (46 mg), 0.17 mmol CsI (44 mg), 0.88 mmol PbI_2 (444 mg, 10% excess of PbI_2), 0.83 mmol FAI (143 mg) in a 1 mL solvent mixture of

DMF:DMSO (4:1 volume ratio). Furthermore, 35 µL of a $PbCl_2$:MACl solution dissolved in 1 mL DMSO with a molar ratio of 1:1 was added to the perovskite precursor solution as a bulk passivation additive. The solution was then deposited via two-step spin coating process: 1) 1000 r.p.m. for 10 s (2000 r.p.m. s^{-1}), 2) 5000 r.p.m. for 40 s (2000 r.p.m. s^{-1}). 20 s before the end of the second spin coating step, 150 µL CB was dropped on the spinning substrate. The deposition of the perovskite absorber was finished by annealing the layer at 100 °C for 30 min in inert atmosphere. After annealing, 1 nm LiF as passivation layer and 23 nm of C60 as electron transport layer were deposited via thermal evaporation at evaporation rates of 0.1-2 Å/s and a pressure of 10^{-6} mbar. This was followed by an atomic layer deposition of 35 nm SnO_2 via 300 cycles. Subsequently, the P2 lines were laser scribed with a laser pulse fluence of 0.4 J/cm^2 and a scribing speed of 33 mm/s to enable a contact between front and back electrode. As transparent back electrode, indium-doped zinc oxide (IZO) with a thickness of 165 nm was deposited via sputtering (Kurt J. Lesker PVD-75). To finish the devices, the P3 lines were laser scribed at a laser pulse fluence of 0.3 J/cm^2 and a scribing speed of 100 mm/s.

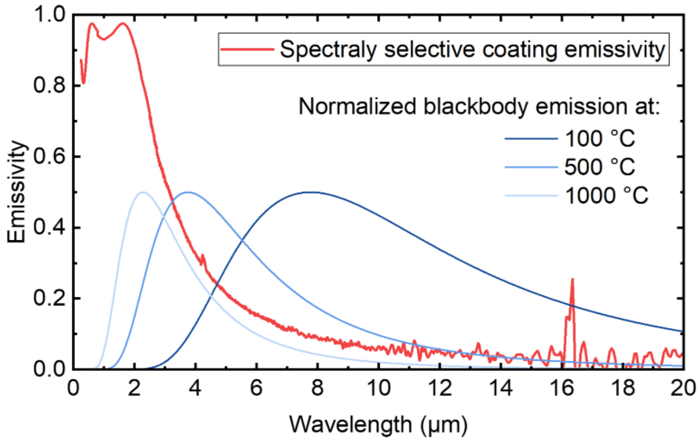

Figure S2. Measured emissivity spectra of the spectrally selective coating of the ST. The background curves show the corresponding normalized blackbody emission spectra at temperatures ranging from 100 °C to 1000 °C.

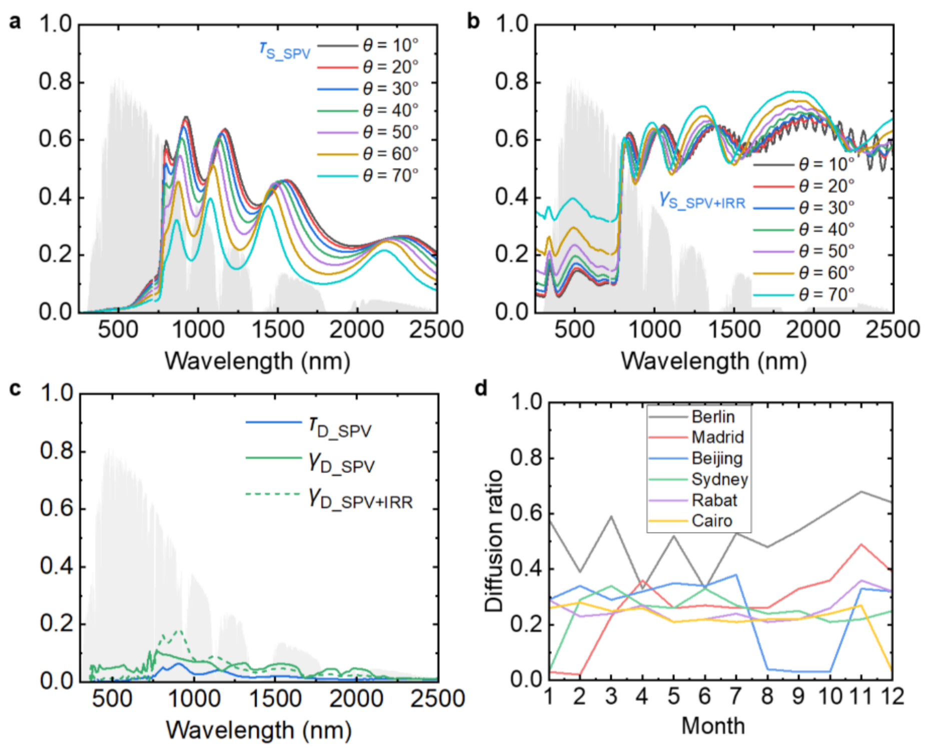

Figure S3. **Additional optical properties of SPV and SPV+IRR. a**, Specular transmittance of SPV (τ_{S_SPV}) for incident angles in the range of 10° to 70°. The transmittance of SPV decreases as the incident angle increases, due to the increase of optical reflection loss. The incident angle has a stronger impact on the transmittance in the out-band range (>780 nm) compared to the in-band range (<780 nm). **b**, Specular reflectance of SPV+IRR ($\gamma_{S\_SPV+IRR}$) for incident angles in the range of 10° to 70°. The incident angle has a stronger impact on the reflectance in the in-band range (<780 nm) compared to the out-band range (>780 nm). **c**, Diffused transmittance and reflectance of SPV (τ_{D_SPV}, γ_{F_SPV}) and diffused reflectance of SPV+IRR ($\gamma_{DF\_SPV+IRR}$). Of note is that the diffused light reflected from SPV+IRR cannot be accurately directed to the ST absorber. **d**, The fraction of diffuse light in different locations for different months[1]. The solar irradiance in Berlin includes a high fraction of diffuse light, averaging around 50%. The solar irradiance in Cairo includes a low fraction of diffuse light, averaging around 20%.

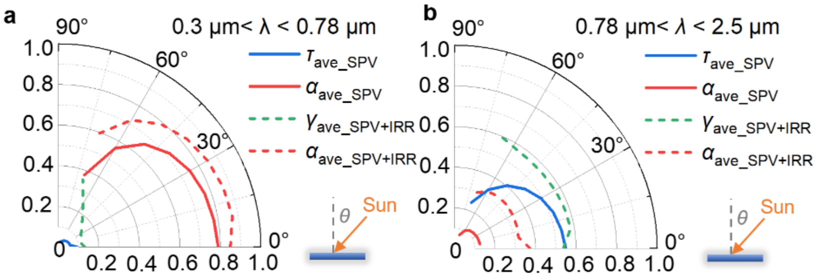

Figure S4. **Spectrally-weighted average optical properties for SPV and SPV+IRR**. **a,** Spectrally-weighted average transmittance and absorptance of SPV, and average reflectance and absorptance of SPV+IRR at wavelength range of 0.3–0.78 µm, which in the in-band spectrum for the perovskite modules. **b,** Spectrally-weighted average transmittance and absorptance of SPV, and average reflectance and absorptance of SPV+IRR at the wavelength range of 0.78–2.5 µm, which in the out-band spectrum for the perovskite modules.

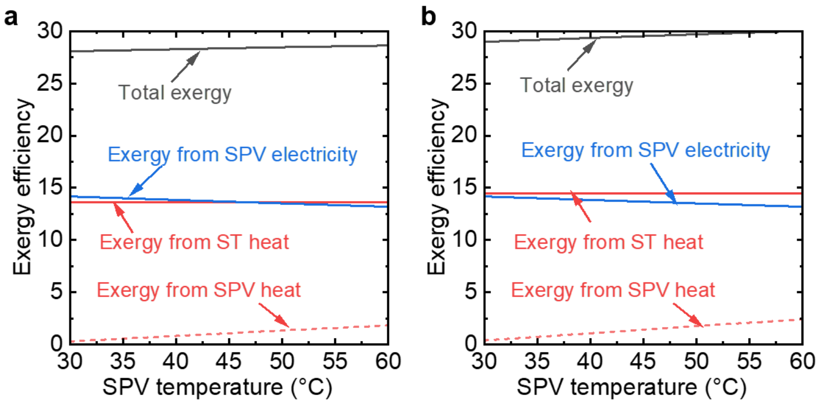

Figure S5. **Impact of the SPV temperature on exergy outputs of (a) PVST-1 system and (b) PVST-2 system.** A lower SPV operating temperature slightly improves power conversion efficiency but reduces the exergy output from SPV heat. This highlights a trade-off between SPV performance and the exergy output from SPV heat. Nevertheless, the impact of SPV temperature on the overall system performance is minimal. Specifically, the overall exergy efficiency of the PVST-1 system decreases only slightly, from 28.6% to 28.3%, when the SPV temperature is reduced from 60 °C to 40 °C. Similarly, the exergy efficiency of the PVST-2 system decreases from 30.0% to 29.5% over the same temperature range. Although a trade-off exists between SPV performance and SPV heat exergy output, the impact of SPV temperature on the total exergy efficiency of the system remains minor. Therefore, the SPV can be cooled further from 60 °C to 40 °C to maintain high exergy output of the system.

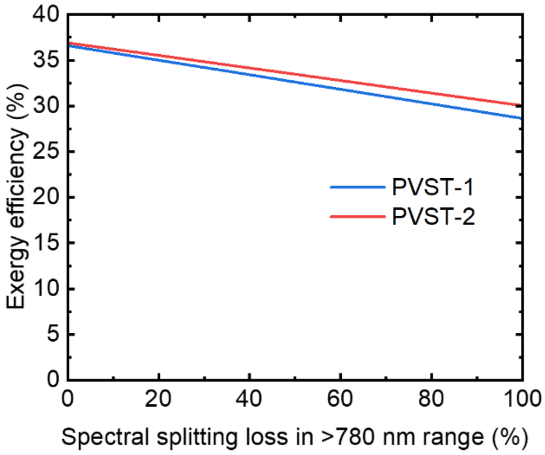

Figure S6. **Impact of spectral splitting loss for wavelengths beyond 780 nm.** In the current module design, a significant portion of the infrared (IR) solar spectrum (wavelengths >780 nm) is either absorbed or reflected by the SPV module, rather than being transmitted to the solar-thermal (ST) absorber. These spectral splitting losses reduce the overall system performance. As shown in Figure S6, the exergy efficiency of PVST-1 could increase from 28.6% to 36.6%, and that of PVST-2 from 30.0% to 36.9%, if these IR losses were completely eliminated under ideal conditions. Even a 50% reduction in these losses would lead to notable improvements, raising the exergy efficiency to 32.6% for PVST-1 and 33.5% for PVST-2.

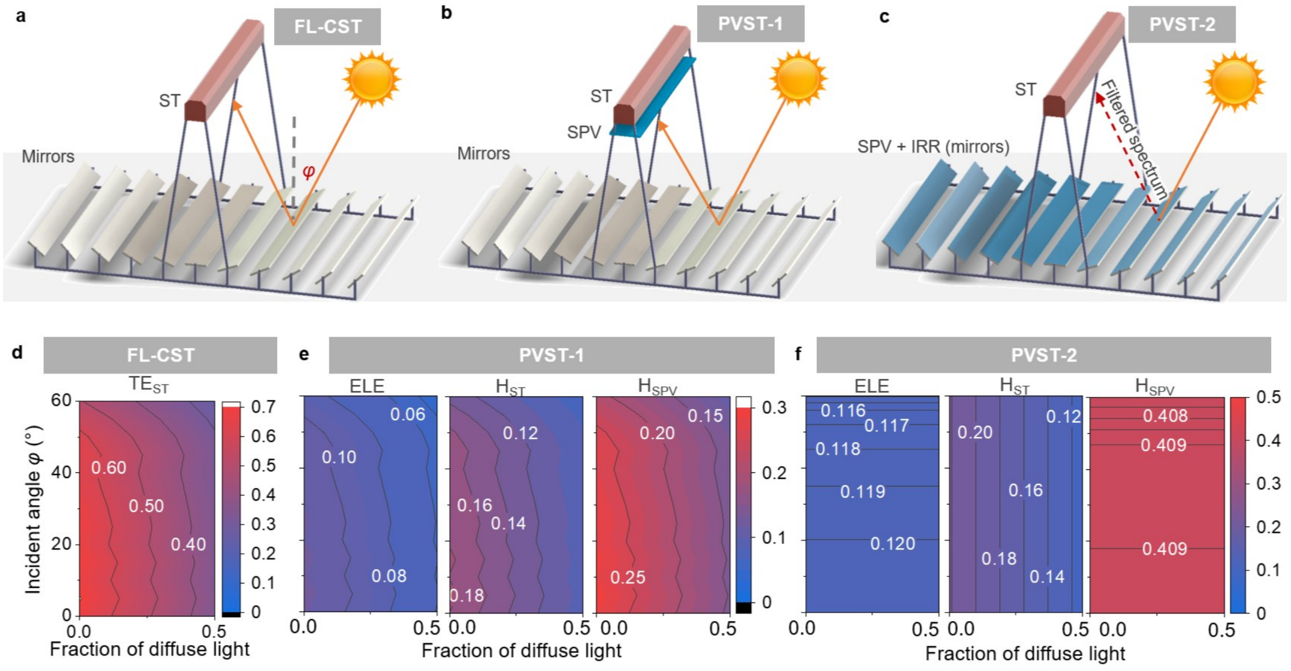

Figure S7. Comparison of the performance of hybrid perovskite-photovoltaic and solar-thermal (PVST) collectors with an existing Fresnel-lens-based concentrated solar thermal (FL-CST) collectors. **a**, A concentrated solar thermal (CST) collector with a Fresnel-lens (FL) solar concentrator[2]. **b**, A hybrid PVST-1 solar collector, which can be upgraded from the FL concentrator by adding the SPV component below the ST collector. **c**, A hybrid PVST-1 solar collector, which has already shown in the main text. **d**. The percentages of solar energy converted to heat in ST (H_{ST}) in various sunlight incident angles (φ) and fraction of diffuse light. The FL concentrator achieves a maximum thermal efficiency of 68% when both the fraction of diffuse light and incident angle are zero[2]. As the incident angle increases, the CST thermal efficiency decreases due to increased optical losses. **e**, The percentages of solar energy converted to electrical power (ELE), heat in SPV (H_{SPV}) and heat in ST (H_{ST}) in various sunlight incident angles (φ) and fraction of diffuse light for the hybrid PVST-1 solar collector. The PVST-1 collector generates electrical power from SPV, thermal energy from ST (H_{ST}) and thermal energy from SPV (H_{SPV}) with efficiencies of 11.8%, 18.0%, and 28.5% respectively at the ambient temperature when fraction of diffuse light is zero and incident angle is zero. The overall efficiency of CPVT-1 is 58.3%, approximately 10% lower than that of the FL concentrator collector. This reduction is due to additional optical losses caused by the SPV component and the cooling channel. **f**, The percentages of solar energy converted to electrical power (ELE), heat in SPV (H_{SPV}) and heat in ST (H_{ST}) in various sunlight incident angles (φ) and fraction of diffuse light for the hybrid PVST-2 solar collector. The PVST-2 collector generate electricity from SPV (ELE), thermal energy from ST (H_{ST}) and heat from SPV (H_{SPV}) with efficiencies of

12.0%, 20.4%, and 42.0% respectively at the ambient temperature when fraction of diffuse light is zero and incident angle is zero, with an overall efficiency of 74.4%, which is significantly higher than those of the FL and PVST-1 collectors. The overall efficiency of FL, PVST-1 and PVST-2 are 54.4% (only heat), 46.7% (9.5% electricity, 22.8% SPV heat and 14.4% ST heat) and 70.7% (12.0% electricity, 40.9% SPV heat and 17.8% ST heat) when the fraction of diffuse light is 20%, indicating that the PVST-2 collector can more efficiently utilise the diffuse light.

The PVST system developed in this study is capable of simultaneously generating electricity and both low- and high-temperature heat, achieving an exergy efficiency of approximately 30%. In contrast, the most common multi-generation solar systems are hybrid photovoltaic-thermal systems, which can generate electricity and low-temperature heat (typically around 60 °C). However, these systems are not able to produce high-temperature heat as in our PVST system, thereby limiting their exergy efficiency to a range of 12% to 23%[3–5].

Perovskite solar cells have also been integrated into double-generation designs in recent years, though with limited heat output temperatures. The low-temperature heat harvested from perovskite-cells can be used directly for heating or converted into additional electricity or cooling. For example, perovskite solar cells incorporated into conventional photovoltaic-thermal collectors can achieve an exergy efficiency of 22% when the heat output temperature is around 60 °C[6]. In other studies, perovskite cells have been combined with thermoelectric generators to convert PV waste heat (approximately 70 °C) into electricity, achieving overall efficiencies between 20% and 23%[7,8]. Similarly, coupling perovskite cells with heat-driven electrocaloric coolers (operating around 72 °C) has resulted in an overall efficiency of 21%[9].

Compared to more advanced double-generation systems using spectral-splitting technologies—such as those based on nanofilms or nanofluids—our PVST design offers significant advantages. While such systems can also produce electricity and both low- and high-temperature heat, they require additional optical filters, which introduce added system complexity[10]. From a theoretical perspective, assuming negligible optical losses, all spectral-splitting photovoltaic-thermal systems should exhibit similar exergy efficiency[11]. However, in practice, substantial and unavoidable optical losses—up to ~50%—have been observed in nanofilm-based spectral-splitting systems[12], which significantly reduce their actual performance. Our PVST system circumvents these losses by eliminating the need for additional optical filters altogether.

With regard to existing tri-generation solar energy systems, which typically deliver electricity, heating, and cooling (often via heat-driven absorption chillers), our current PVST system is not yet designed to produce cooling and therefore cannot be directly compared to such

configurations. A representative case study on hybrid solar systems for buildings in Bari, Italy, reported that a tri-generation solar system was able to meet 18%, 58%, and 22% of building demands for electricity, cooling, and heating, respectively[13]. Nevertheless, we note that absorption chillers generally operate more efficiently when driven by high-temperature heat sources[14]. Given that our PVST system can deliver high-temperature heat, it holds promising potential for future extension into tri-generation applications with enhanced cooling capabilities.

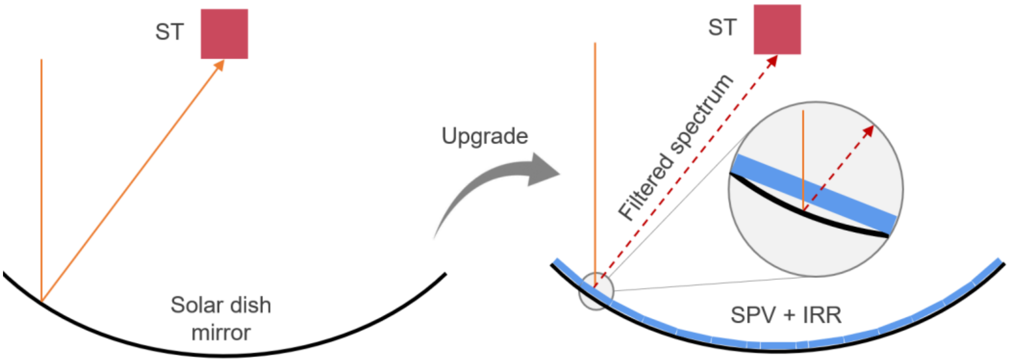

Figure S8. **A hybrid PVST collector based on the PVST-2 concept**. The hybrid solar collector can be upgraded from the solar dish (SD) solar concentrator by attaching the SPV cells above the minors of the SD. The small SPV cells can be paved onto the solar dish mirror. There light can pass through the flat SPV and then be reflected by the curved IRR.

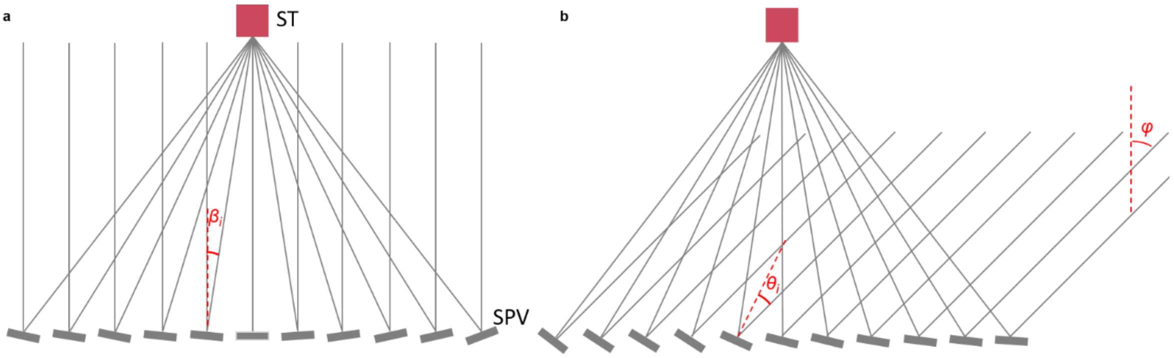

Figure S9. **Sunlight ray and angles in the Fresnel-lens (FL) concentrators**. **a**, Ray distribution when the incident sunlight is normal to the ground. The angle between the incident light and reflection light is β_i for each mirror *i*. **b**, Ray distribution when the incident sunlight has an angle of φ with the vertical direction. The incident angle for a mirror is θ_i, which can be calculated by $\theta_i = (\varphi - \beta_i)/2$. The incident angle for each mirror (SPV+IRR) can thus be precisely calculated for a given φ. The modelling thus can utilise the optical and electrical properties of SPV with different incident angles to calculate the performance and efficiencies.

References

1. Photovoltaic Geographical Information System (PVGIS), https://re.jrc.ec.europa.eu/pvg_tools/en/ [Access date: 08 February 2024].
2. Industrial Solar GmbH, Fresnel Collector LF-11 Datasheet, https://industrial-solar.de/wp-content/uploads/2022/06/LF-11_Technical-Datasheet_EN.pdf [Access date: 08 February 2024].
3. Alkhalidi, A., Salameh, T., & Al Makky, A. (2024). Experimental investigation thermal and exergy efficiency of photovoltaic/thermal system. Renewable Energy, 222, 119897.
4. Aberoumand, S., Ghamari, S., & Shabani, B. (2018). Energy and exergy analysis of a photovoltaic thermal (PV/T) system using nanofluids: An experimental study. Solar Energy, 165, 167-177.
5. Sardarabadi, M., Passandideh-Fard, M., Maghrebi, M. J., & Ghazikhani, M. (2017). Experimental study of using both ZnO/water nanofluid and phase change material (PCM) in photovoltaic thermal systems. Solar Energy Materials and Solar Cells, 161, 62-69.
6. Li, X., Chen, K., Su, J. M., Zhou, H., Ren, Z., Zhao, B., & Pei, G. (2025). Performance study of a vacuum photovoltaic/thermal collector with spectral selectivity. Renewable Energy, 239, 122130.
7. Xu, L., Xiong, Y., Mei, A., Hu, Y., Rong, Y., Zhou, Y., ... & Han, H. (2018). Efficient perovskite photovoltaic-thermoelectric hybrid device. Advanced Energy Materials, 8(13), 1702937.
8. Liao, T., He, Q., Xu, Q., Dai, Y., Cheng, C., & Ni, M. (2020). Performance evaluation and optimization of a perovskite solar cell-thermoelectric generator hybrid system. Energy, 201, 117665.
9. Lu, Z., Huang, Y., & Zhao, Y. (2023). Elastocaloric cooler for waste heat recovery from perovskite solar cell with electricity and cooling production. Renewable Energy, 215, 118972.
10. Huang, G., Curt, S. R., Wang, K., & Markides, C. N. (2020). Challenges and opportunities for nanomaterials in spectral splitting for high-performance hybrid solar photovoltaic-thermal applications: a review. Nano Materials Science, 2(3), 183-203.
11. Huang, G., Wang, K., & Markides, C. N. (2021). Efficiency limits of concentrating spectral-splitting hybrid photovoltaic-thermal (PV-T) solar collectors and systems. Light: Science & Applications, 10(1), 28.

12. Todd Otanicar, John Dale, Matthew Orosz, Nick Brekke, Drew DeJarnette, Ebrima Tunkara, Kenneth Roberts, and Parameswar Harikumar (2018). Experimental evaluation of a prototype hybrid CPV/T system utilizing a nanoparticle fluid absorber at elevated temperatures. Applied Energy, 228, 1531-1539
13. Herrando, M., Pantaleo, A. M., Wang, K., & Markides, C. N. (2019). Solar combined cooling, heating and power systems based on hybrid PVT, PV or solar-thermal collectors for building applications. Renewable Energy, 143, 637-647.
14. Shirazi, A., Taylor, R. A., Morrison, G. L., & White, S. D. (2018). Solar-powered absorption chillers: A comprehensive and critical review. Energy conversion and management, 171, 59-81.
